# Outcomes of physical exercises on initiation, progression, and treatment of breast cancer

**DOI:** 10.1186/s12964-024-01634-6

**Published:** 2024-05-07

**Authors:** Junwei Xu, Xiance Jiao, Reyhaneh Bayat

**Affiliations:** 1 Physical education institute, Henan University of Economics and Law, Zhengzhou, China; 2 College of Continuing Education, Henan Quality Polytechnic, Pingdingshan, China; 3 Department of General Surgery, Shahid Beheshti University of Medical Science and Health Services, Taleghani Hospital, Tehran, Iran

**Keywords:** Breast cancer, Physical exercises, Cancer therapy, Resistance

## Abstract

The emergence of drug resistance is a substantial obstacle to the effective management of breast cancer, which is the primary cause of cancer-related deaths in women worldwide. To facilitate the development of targeted therapies that can effectively overcome drug resistance, it is crucial to possess a comprehensive comprehension of the molecular mechanisms that underpin resistance to breast cancer treatment. So far, considerable progress has been made in the field of exercise-oncology research and overcome drug resistance, specifically about breast cancer. Evidence has suggested that participation in physical activity is correlated with a decrease in reappearance and fatality rates of breast cancer patients. It has been reported that participation in physical activity can yield favorable outcomes in the prevention, treatment, and post-treatment of breast cancer. An increasing body of empirical evidence suggests that participation in physical activity can alter diverse biological mechanisms, potentially augmenting breast cancer treatments’ efficacy. Comparing increased physical activity versus reduced physical activity in breast cancer patients who received chemotherapy, radiotherapy, and surgery supported the significance of exercise in comprehensive care strategies to enhance overall health and treatment efficacy. Furthermore, previous studies have reported that physical activity can enhance the efficacy of breast cancer treatments. This review provides the current literature regarding the influence of physical activity on the occurrence and progression of breast cancer.

## Introduction

Breast cancer is a common malignancy in women worldwide, with an estimated 2.3 million new cases projected for the year 2020. This figure represents approximately 24.5% of the total number of cancer diagnoses [1]. The median survival rate for metastatic breast cancer, characterized by the presence of breast cancer in distant sites from the breast and lymph nodes, has demonstrated a substantial rise from 4 to 30% within the previous two decades [2]. The therapeutic objectives for metastatic breast cancer encompass extension of life expectancy, increase of quality of life, and management of symptoms [3, 4]. The determination of appropriate treatment modalities for metastatic breast cancer is contingent upon a multitude of factors, including precise anatomical site and morphological characteristics of the tumor. The current treatment approaches are radiotherapy, hormone blockade, immunotherapy, and chemotherapy [5]. Although it is widely recognized that these interventions enhanced survival rates, it is crucial to acknowledge that these approaches may also associated with various adverse outcomes [6]. Nevertheless, the emergence of drug resistance poses a substantial barrier to the efficacy of breast cancer treatment. It continues to be the primary factor contributing to cancer-related fatalities in the world [7].

**Fig. 1:**
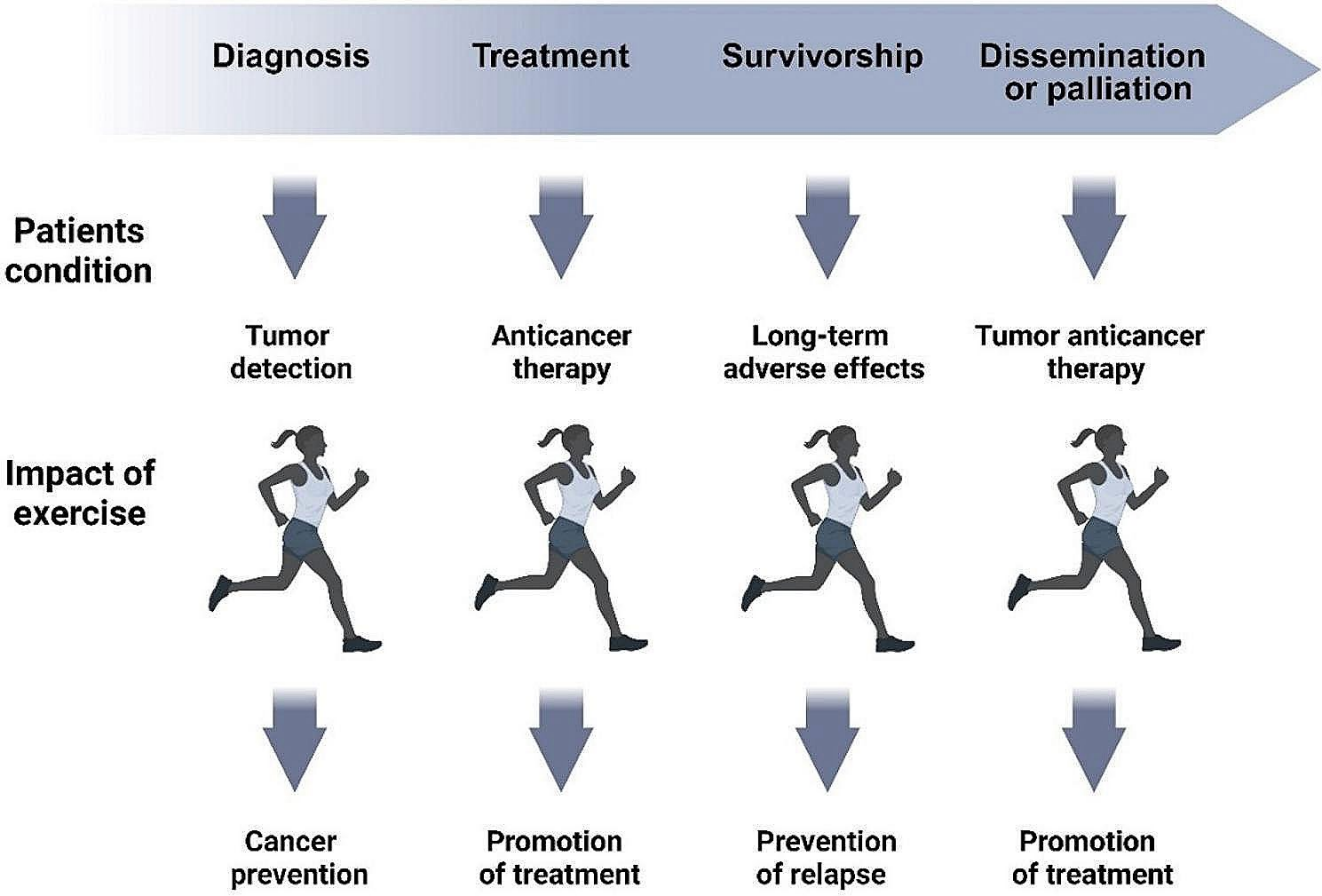
The influence of physical activity on the progression of cancer. The cancer care continuum comprises multiple stages encompassing the identification and diagnosis of cancer, the implementation of suitable treatment strategies, the provision of support for cancer survivors, the management of disease progression, and the delivery of palliative care. Distinct pathological conditions are associated with each stage of the cancer continuum. These conditions encompass the presence of tumors, the utilization of anti-cancer therapy, the manifestation of long-term adverse effects, and the intricate interplay among these factors. Exercise training possesses the ability to accomplish diverse objectives across this continuum. The intervention possesses the capacity to generate a multitude of benefits. These benefits encompass a decreased probability of cancer manifestation in the pre-diagnostic phase, improved tolerability and efficacy of pharmacological interventions, lowered risk of cancer recurrence, alleviation of adverse effects associated with initial anti-cancer therapy, reduced likelihood of concurrent development of other medical conditions, and promotion of post-treatment recovery of physical function and overall well-being

According to compelling empirical data, a notable association has been established between heightened levels of physical activity and a decreased incidence of premenopausal and postmenopausal breast cancer [8, 9]. Participating in physical activity significantly improves cardiopulmonary function, psychological well-being, muscular strength, and endurance in individuals who have effectively conquered breast cancer [10, 11]. Based on estimations, individuals who have been diagnosed with breast cancer exhibit a reduction of approximately 11% in their physical activity levels, underscoring the significance of these benefits [12]. A greater percentage of breast cancer survivors who undergo chemotherapy (50%) and radiation therapy (24%) demonstrate a more significant decline in physical activity levels compared to those who do not undergo these treatments (12%). Engaging in higher levels of physical activity can yield significant effects, such as reducing body mass and altering body composition. These outcomes are widely acknowledged as risk factors associated with the development of breast cancer [2]. The investigation of exercise post-breast cancer treatment is of great importance due to the numerous benefits associated with physical activity, as well as the substantial increase in activity levels following a diagnosis of cancer. Effects of physical exercises in the cancer continuum (detection, treatment, survivorship, and disease dissemination and palliation) are presented in Fig. 1.

Several studies have conducted comprehensive analyses to investigate the impact of various forms of physical activity on the therapeutic outcomes of individuals diagnosed with any form of cancer, encompassing breast cancer patients across all stages of the ailment [13, 14]. Nevertheless, the currently available literature on the association between physical activity and individuals who have been diagnosed with breast cancer, currently there is a significantly limitations in the exact mechanisms of this scope. This study aimed to assess and consolidate the current body of evidence concerning the beneficial impact of physical activity on the effectiveness of breast cancer treatment.

## Association of physical exercises and breast cancer

Physical activity plays a pivotal role in facilitating a range of physiological and biological mechanisms that govern the progression of different types of cancer. Moreover, it is crucial to recognize that the manifestation of these reactions is impacted by systemic inflammation and metabolic and hormonal alterations [15]. Numerous recent studies have provided evidence of a noteworthy correlation between the ability to influence tumor progression directly and changes in tumor vascularization and blood flow, the metabolic utilization of substrates by malignant cells, the protein-level interactions between cancer cells and muscle tissue, and the modulation of immune function through physical exercise [16, 17]. Extended periods of physical activity induce metabolic and immunogenic changes that hinder cancer progression [18, 19].

Multiple research studies have provided evidence suggesting that women who partake in regular physical exercise exhibit a decreased susceptibility to breast cancer development, with risk reductions ranging from 10 to 25%, in comparison to women who do not adhere to a consistent exercise routine [20, 21]. The correlation under observation appears to exhibit greater prominence within the female population characterized by a normal body mass index (BMI), the absence of familial susceptibility to breast cancer, and a history of at least one childbirth. The existing literature on the influence of physical activity on breast cancer prevention in individuals with BRCA1 and BRCA2 mutations is constrained, thereby highlighting the requirement for more extensive investigations to explore this association in greater detail [22, 23]. Moreover, previous research has indicated a positive correlation between participation in physical activity and a reduction in the likelihood of developing breast cancer. Individuals who participate in elevated levels of physical activity demonstrate a heightened propensity for experiencing a significant decrease in their susceptibility to the development of breast cancer [24, 25].

**Fig. 2:**
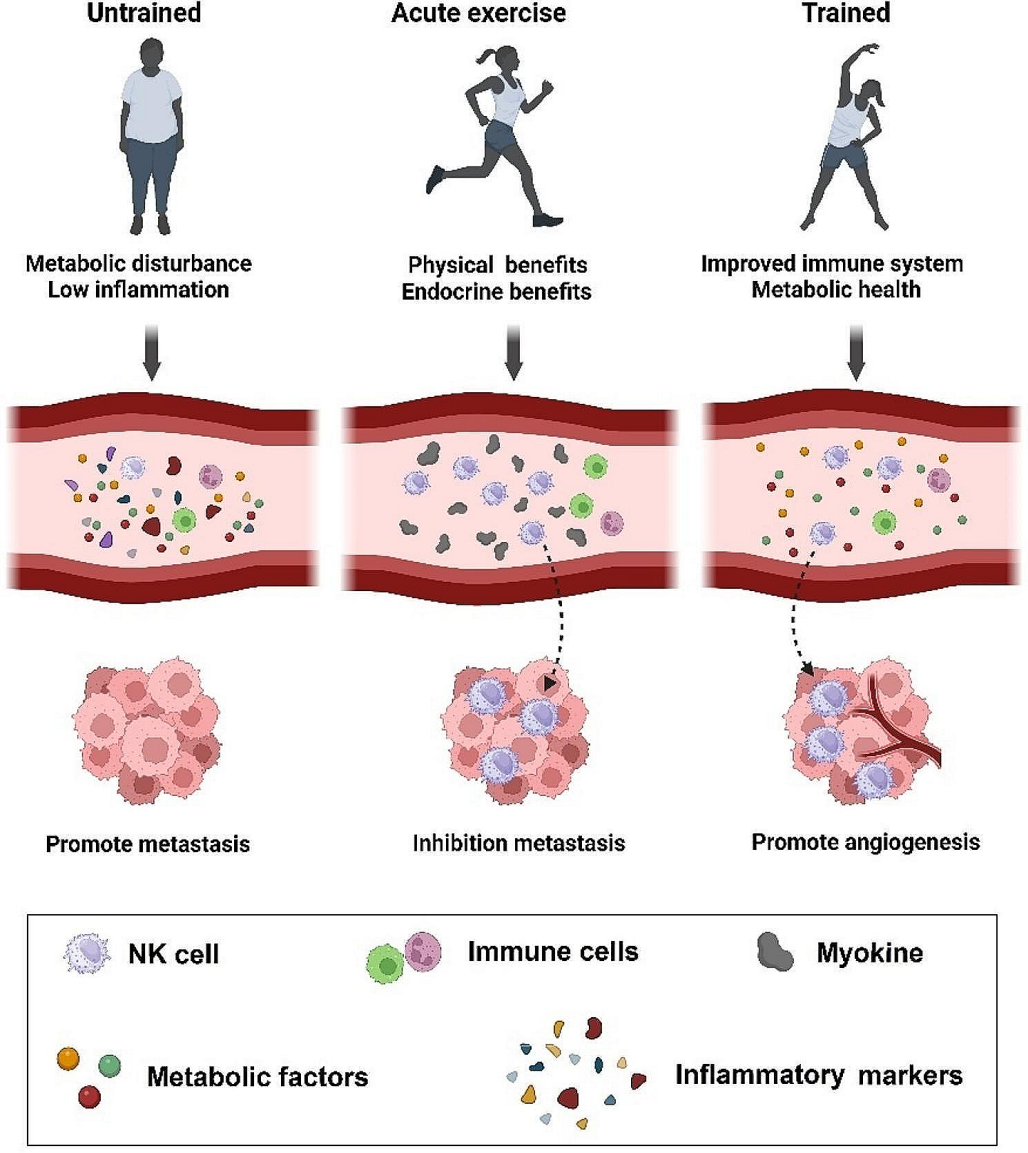
A well-documented relationship has been established between the participation in physical exercise and the potential reduction of cancer risk, with this connection being attributed to underlying molecular mechanisms. Prior research has indicated that brief periods of intervention possess the capacity to augment the blood flow to tumors, facilitate the transportation of oxygen, alleviate metabolic stress within the tumor, mitigate cellular damage, and promote the production of reactive oxygen species. The abrupt modifications possess the capacity to initiate signaling cascades that impede the progression of metastasis. Chronic training adaptations are characterized by the enhancement of immune function, the reduction of systemic inflammation, and the improvement of metabolic health. Intratumoral alterations can be distinguished by heightened blood perfusion, an immunogenic profile, and enhanced infiltration of immune cells. There exists a documented correlation between the participation in physical exercise and the potential mitigation of cancer, wherein this association is connected to fundamental molecular mechanisms. Previous studies have indicated that acute sessions have the potential to enhance tumor perfusion, oxygen delivery, intratumoral metabolic stress, cellular injury, and the generation of reactive oxygen species. The sudden alterations have the ability to initiate signaling cascades that hinder the advancement of metastasis. Chronic training adaptations are distinguished by the augmentation of immune function, mitigation of systemic inflammation, and amelioration of metabolic health. Intratumoral modifications are characterized by increased blood perfusion, an immunogenic profile, and augmented infiltration of immune cells

Empirical evidence indicates that participation in physical activity can diminish estrogen activity, insulin resistance, inflammation, and oxidative stress [26]. Research has demonstrated that estrogen can exert inhibitory effects on cellular division and the progression of tumor formation. Research has demonstrated that engaging in physical activity can augment globulin synthesis. This protein exhibits an affinity for reproductive hormones, leading to a subsequent decrease in the levels of estrogen present in the circulatory system. This intervention’s primary contribution is to reduce adipose tissue mass, specifically targeting visceral fat. This intervention also enhances cellular insulin sensitivity, leading to a subsequent decrease in serum insulin levels. Furthermore, it is important to acknowledge that participation in physical activity has been discovered to possess immunomodulatory characteristics. This encompasses the capacity to augment intrinsic and acquired immune responses and mechanisms for repairing DNA. As a result, these effects play a role in decreasing the probability of breast cancer development [26, 27]. Additional research is required to acquire a thorough comprehension of the mechanisms by which physical activity reduces the likelihood of developing breast cancer. Potential molecular mechanisms linking physical exercises to cancer protection are presented in Fig. 2.

## Association of physical exercises and breast cancer metastasis

According to research findings, engaging in regular moderate-intensity physical activity has been shown to potentially hinder the initiation of a metastatic cascade and the subsequent development of cancer metastases. The results above are achieved through the regulation of angiogenesis, the elimination of circulating tumor cells, and the reduction of endothelial cell permeability [28]. On the other hand, evidence suggests that engaging in intense physical activity may contribute to the proliferation of cancer cells. The use of animal models does not produce definitive outcomes. For instance, in a rodent model of breast cancer, it has been observed that engaging in moderate-intensity exercise training generally provides a protective effect against the formation of pulmonary metastases [29]. Nevertheless, a preclinical study conducted on rodents suggests that engaging in four weeks of wheel running following the introduction of breast cancer cells is correlated with a higher incidence of pulmonary metastases in comparison to sedentary control subjects. Moreover, this exercise regimen has been associated with reducing nitric oxide production [30]. Nevertheless, a preliminary clinical intervention was implemented on a specific group of individuals who had received a diagnosis of breast cancer. The intervention was a 12-week protocol incorporating aerobic exercise training and neoadjuvant chemotherapy. The findings of this intervention revealed a significant augmentation in the production of nitric oxide and a notable decrease in the invasiveness of the tumor [31].

Participating in physical exercise facilitates the advancement of the M1 phenotype within tumor-associated macrophages (TAMs), thereby enhancing their capacity to counteract tumors efficiently. TAMs can differentiate into two distinct phenotypes: M1, characterized by its capacity to exert anti-tumor effects, and M2, recognized for facilitating tumor growth, local invasion, metastasis, and immune suppression. Furthermore, there is empirical evidence suggesting that the involvement in moderate to vigorous physical activity impedes the recruitment of macrophages to the tumor microenvironment (TME) in instances of breast cancer. Conversely, engaging in inactive behaviors can facilitate the formation of the M2 phenotype, thus promoting tumor growth, invasion, and metastasis [32].

**Fig. 3:**
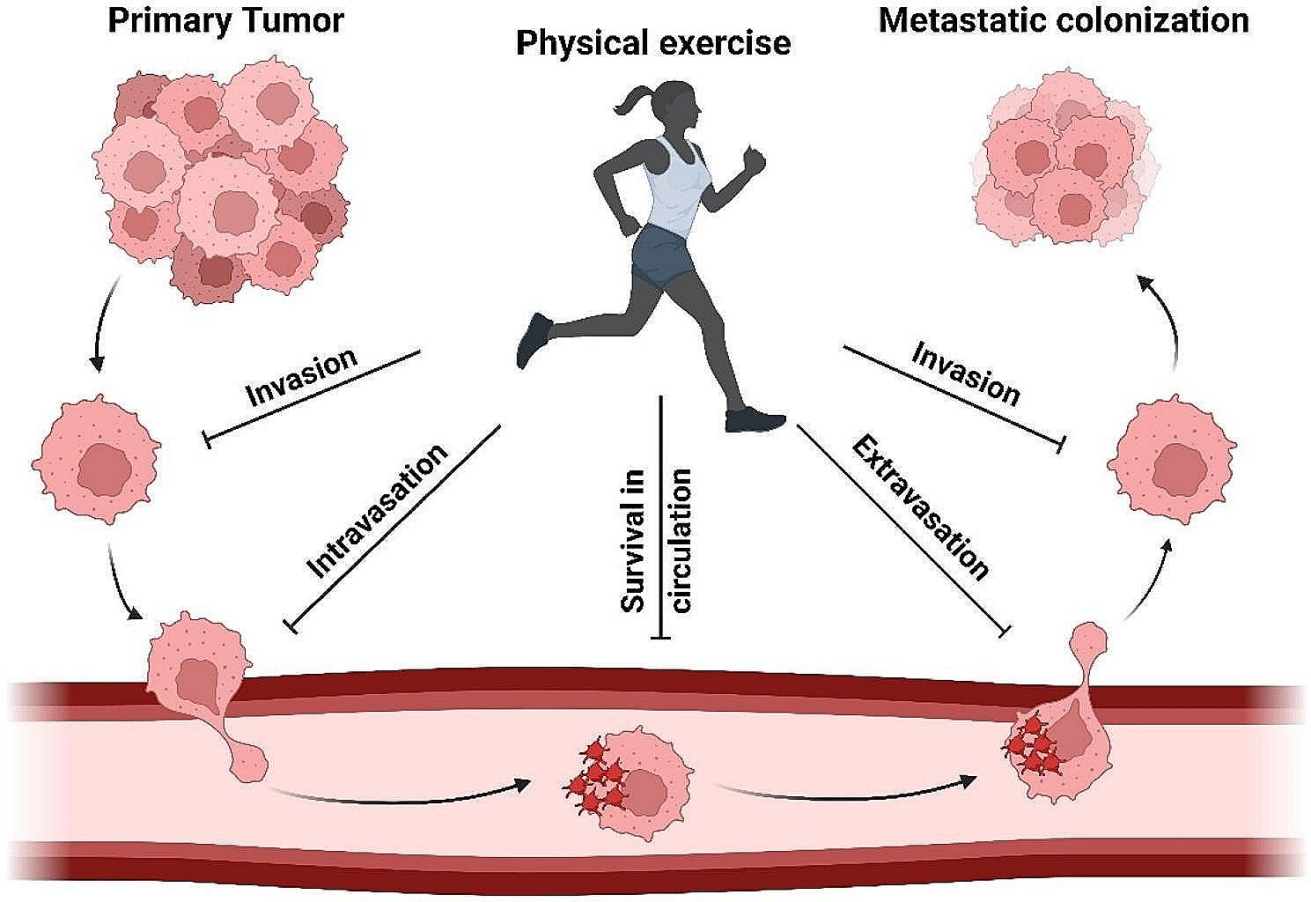
The relationship between physical activity and the progression of metastatic disease. The initial results indicate that participation in physical activity is linked to a reduction in the invasion of tumor cells by inhibiting the epithelial-mesenchymal transition (EMT) mechanism. Consequently, participation in physical activity resulted in a decrease in vascular permeability, inhibition of the interaction between endothelial cells and tumor cells, and a reduction in intravasation. Furthermore, aside from enhancing vascular shear force, mobilizing macrophages, natural killer (NK) cells, and CD8 + T cells, regulating metabolism, and inducing apoptosis, physical activity possesses the capability to impede the viability of circulating tumor cells (CTCs). The findings of the study indicate that participation in physical activity can effectively impede the formation of platelet-tumor cell aggregates and the adherence of tumor cells to endothelial cells. These phenomena have been identified as factors that hinder the extravasation process

The correlation between tumor invasion and metastasis has been observed in connection with the secretion of irisin, a myokine, during physical exertion. Prior research has documented a decrease in the concentration of Irisin in the bloodstream of individuals who have received a breast cancer diagnosis [33, 34]. However, previous studies have provided evidence indicating that engagement in physical activity can lead to an elevation in the levels of Irisin in the circulatory system, potentially contributing to the prevention of spinal metastasis [35, 36]. The act of participating in physical activity has been observed to stimulate the release of anti-metastatic adipokines, such as adiponectin, while simultaneously inhibiting the synthesis of pro-metastatic adipokines, such as leptin and tumor necrosis factor-alpha (TNF-α) [37, 38]. Moreover, substantial evidence indicates that engagement in physical activity can increase the expression of myomiR-133a, a distinct subtype of miRNA. The miRNA in question is implicated in the mechanism of myoblast differentiation and has been observed to play a role in suppressing the invasion and metastasis of breast cancer tumors [39, 40]. The association of physical exercises and metastatic cascade in breast cancer is presented in Fig. 3.

## Association of physical exercises and breast cancer angiogenesis

Previous experimental studies conducted in a murine model of breast cancer at a specific anatomical site have indicated that physical exercise promotes angiogenesis within the tumor and enhances the density of microvasculature [41]. The observation above is supported by a reduction in hypoxia levels within the tumor and an augmentation in tissue perfusion, oxygen availability, and the presence of nitric oxide [42, 43]. As a result, these alterations give rise to an adverse milieu for the advancement of cancer and enhance the administration of therapeutic medications [44]. In a subsequent preclinical investigation involving mice, it was observed that endurance exercise reduced the expression of vascular endothelial growth factor-A (VEGF-A) in tissue samples [45].

On the other hand, previous studies have demonstrated that a meticulous training protocol for mammary tumors in rodents effectively confirms the enhancement of VEGF-A expression and the development of tumor vasculature [46]. Based on findings from a clinical study, there exists a proposed association between an augmentation in the total duration of physical activity and the reduction of body weight, resulting in a decline in the manifestation of circulating VEGF-A in women diagnosed with breast cancer who are classified as obese [47]. Nevertheless, it is important to acknowledge that the effectiveness of a comprehensive training intervention, which incorporates both aerobic and resistance training, following surgical procedures, radiotherapy, or chemotherapy, lacks substantiating evidence [48]. However, the current body of evidence concerning the correlation between physical activity and breast cancer angiogenesis is limited, highlighting the need for further investigation to enhance our comprehension of this topic.

## Physical exercises and breast cancer prevention

Physical activity encompasses diverse bodily movements the skeletal muscles execute, resulting in energy expenditure [49]. The correlation between physical activity and heightened global DNA methylation underscores the notable significance of physical activity in preventing breast cancer [50]. A considerable body of empirical research suggests that a noteworthy percentage, ranging from 90 to 95%, of cancer cases can be ascribed to lifestyle and environmental factors. The discovery above underscores the potential of mitigating cancer risk through adherence to recommended dietary practices, regular physical activity engagement, and effective body weight management, as delineated in established guidelines [51]. Based on previous studies, there exists a hypothesis suggesting that the adoption of efficacious lifestyle management strategies possesses the potential to reduce approximately 25% of breast cancer incidences. Prior research has presented empirical findings suggesting that women who engage in regular physical activity exhibit a diminished probability of acquiring breast cancer, with a reduction of approximately 25% compared to women who maintain a sedentary way of life [52, 53].

Based on the available empirical evidence, it can be deduced that premenopausal women benefit more from physical exercise than postmenopausal women. Prior research has suggested that postmenopausal women might need higher physical activity levels to attain similar outcomes [54, 55]. Based on the available literature, it can be deduced that premenopausal women who have a familial predisposition to breast cancer may experience a comparatively reduced benefit from engaging in physical activity, in contrast to postmenopausal women [56]. An individual’s age significantly impacts the relationship between engagement in physical activity and the reduction of breast cancer risk. Hence, based on empirical evidence, it can be inferred that physical activity provides a protective advantage of approximately 16% during adolescence, 8% during early adulthood, 15% during middle adulthood, and 17% for females aged 50 and above [57].

The observed rise in breast cancer occurrences among women below the age of 40 raises concerns about potential consequences [58]. It is important to recognize that the potential advantages of engaging in physical exercise may be diminished for this particular demographic, underscoring the importance of identifying and integrating supplementary preventive strategies alongside physical exercise for this population. Moreover, the early stages of life represent a critical period of vulnerability in mitigating the risk of developing breast cancer, specifically among individuals aged 5 to 19 [59]. Lammert et al. observed a significant reduction of 38% in the vulnerability to breast cancer in adolescents aged 12 to 17 years who carry the BRCA1/2 gene mutation and engage in regular physical activity [60]. On the other hand, a subsequent investigation revealed that this correlation was observed solely in females who experienced a prolonged average duration of 20 years between the onset of menstruation and the initiation of pregnancy. This discovery highlights the conflicting nature of the existing evidence regarding the influence of physical activity on the vulnerability to breast cancer [61].

In a notable study conducted by Kyu et al., it was observed that females who engaged in varying levels of physical activity, namely moderate, high, and very high, experienced a proportional reduction in their vulnerability to breast cancer, with decreases of 3%, 6%, and 14% respectively [62]. According to the study conducted by Chan et al., premenopausal women exhibited a higher level of responsiveness towards overall, leisure, and work-related physical activities than premenopausal women [9]. There is a notable disparity in the inclination towards engaging in physically demanding activities between premenopausal and postmenopausal women. A positive correlation has been observed between the dosage or intensity of physical activity and the level of protection against breast cancer. Nevertheless, individuals must comply with the minimum recommendations [9]. 

## Physical exercises and breast cancer treatment

The evidence suggests that participating in moderate to vigorous physical activity benefits both general mortality rates and specific causes of mortality. Additionally, initial investigations indicate a potential association between engagement in moderate to vigorous physical activity and a reduced probability of developing breast cancer during the course of treatment [63]. The contemporary approach to the management of breast cancer encompasses a comprehensive strategy that effectively integrates both local and systemic therapeutic interventions. Local therapy encompasses a range of interventions, such as surgical procedures, radiotherapy, and breast reconstruction, that are focused on the specific area affected. On the other hand, systemic therapy involves broader treatment approaches, including chemotherapy, hormone therapy, and biological therapy (immunotherapy and target therapies), which target the entire body rather than a localized region [48]. Chemotherapy has been linked to a range of negative effects, such as fatigue, loss of appetite, low red blood cell count, low white blood cell count, low platelet count, peripheral nerve damage, and, to a lesser extent, damage to the heart [64, 65].

Hormone therapy has been linked to a range of adverse outcomes, including but not limited to weight gain, joint pain, muscle pain, decreased bone density, cardiovascular effects, and changes in lipid levels [66]. Furthermore, it is crucial to recognize that radiation therapy has the potential to induce lymphedema, brachial plexopathy, and secondary malignancies, in addition to the well-documented adverse effects on cardiac and pulmonary health [67]. The disease can significantly impact a prominent expression of femininity, sexuality, and maternity, resulting in emotional changes such as sadness, anxiety, decreased self-esteem, and a negative evaluation of one’s physical appeal [68]. Participation in physical activity is widely acknowledged as a secure practice that can be undertaken throughout different stages of cancer treatment. The practice above has yielded beneficial outcomes regarding the overall quality of life, functional capacities, and psychological well-being of individuals undergoing cancer treatment [69, 70].

A considerable percentage of individuals who have been diagnosed with breast cancer commonly report the occurrence of pain. Based on empirical evidence, a significant proportion of individuals seeking medical treatment, ranging from 30 to 60%, experience degrees of pain that can be categorized as moderate to severe. Pain detrimentally influences individuals’ ability to engage in routine activities and limits their physical capabilities, both during therapeutic interventions and in the subsequent period [71, 72]. The extant empirical evidence suggests that physical training directly influences diverse facets of human physiology. The benefits encompass enhancements in physical strength, augmented capacity of the cardiorespiratory system, heightened flexibility, reductions in fatigue, duration of hospitalization, levels of anxiety and depression, sleep disturbances, and symptoms such as vertigo and vomiting [73, 74].

Previous research has established a favorable correlation between resistance exercise interventions and enhancements in self-esteem, muscular strength, and body composition in individuals undergoing chemotherapy. Research findings indicate that exercise programs do not affect patients’ lymphedema or other post-surgical complications. Incorporating three discrete exercise protocols, specifically aerobic, resistance, and flexibility training, has effectively mitigated pain and fatigue symptoms. The observed improvement in cardiorespiratory function in combined exercise training programs can be attributed to the synchronization of ventilation-perfusion and the oxidative capacity of skeletal muscle [75, 76].

The alignment above can result in an augmentation of aerobic capacity, as evidenced by maximal oxygen consumption. The factor mentioned above has the potential to greatly influence the management of structural abnormalities resulting from the harmful consequences of chemotherapy and radiation therapy. Chemotherapeutic agents exhibit both direct and indirect cardiotoxic effects, thus playing a role in the hastening of overall and vascular aging mechanisms, ultimately leading to a reduction in cardiopulmonary capacity [77]. The findings above highlight the significance of integrating physical activity as an additional therapeutic approach in the management of breast cancer, particularly in alleviating the adverse consequences linked to the condition.

## Physical exercises and breast cancer post-treatment

Chronic complications, such as a deterioration in health-related quality of life (HRQoL), diminished physical fitness, and changes in body composition, may hinder the recuperation and progress of individuals diagnosed with breast cancer following curative treatment [78, 79]. The psychosocial and physiological well-being of cancer survivors is adversely impacted by the deleterious effects of treatment, resulting in a diminished overall quality of life [80, 81]. Each therapeutic modality, including surgical interventions (such as radical or partial mastectomy, with or without reconstruction, and with or without lymph node dissection), radiotherapy, and various systemic treatment approaches (such as chemotherapy, hormone therapies, and targeted therapies), is associated with specific adverse effects that have the potential to impact HRQoL [82] adversely. Based on the existing body of evidence, it can be inferred that participation in physical activity has a beneficial effect on the HRQoL of individuals who have successfully overcome breast cancer. There is a consensus among scholars that adhering to the prescribed frequency of physical activity sessions, typically two to three times per week has been substantiated by empirical evidence to improve HRQoL and overall health status [83, 84]. However, it is crucial to recognize that the current body of evidence regarding this topic is somewhat limited, consisting of studies with varying levels of quality, ranging from low to moderate.

Cardiorespiratory endurance and muscular strength are essential components that form the foundation of physical fitness. The quantification of maximum oxygen consumption (VO_2_ max) is a reliable method for accurately assessing impairment related to cardiovascular disease. This measure serves as a dependable indicator of an individual’s cardiorespiratory fitness. Moreover, prior research has presented empirical findings suggesting that the magnitude of this specific factor is reduced in individuals who have successfully overcome breast cancer, as opposed to women who exhibit optimal physical well-being. Furthermore, it is important to acknowledge that this reduction in decline carries significant significance following the introduction of post-adjuvant therapy and is impacted by a range of factors [85]. Breast cancer survivors frequently exhibit risk factors linked to cardiovascular disease and tend to adopt a high-risk lifestyle, which is characterized by sedentary behavior [86, 87].

In addition, cardiotoxicity presents a substantial barrier in the realm of clinical practice [87, 88]. The complication above is commonly observed in the context of conventional cytotoxic therapies employed for breast cancer, such as anthracyclines and HER2-targeted treatments [89, 90]. Participating in physical activity is highly significant for individuals suffering from cardiovascular disease as a fundamental element of their cardiac rehabilitation program. Moreover, empirical research has demonstrated a correlation between participation in physical activity and a reduced risk of developing chronic illnesses, particularly cardiovascular diseases [91]. The existing body of evidence regarding the influence of physical activity on the cardiovascular well-being of breast cancer survivors is presently insufficient.

According to prior studies, a notable proportion of individuals who have effectively surmounted breast cancer have reported experiencing considerable levels of fatigue throughout and after their chemotherapy regimen [92, 93]. Cancer-related fatigue often triggers a detrimental cycle characterized by diminished physical activity due to fatigue, leading to a notable decline in muscle mass and strength in both the upper and lower extremities [94]. A significant association has been observed between decreased values and increased mortality rates across multiple causes, including breast cancer, particularly as reported in studies [95, 96]. The assessment of maximum handgrip strength is a reliable indicator for evaluating overall muscular strength. Recent meta-analytic studies have yielded empirical evidence that supports the effectiveness of exercise in alleviating cancer-related fatigue, both during treatment and in the post-treatment phase.

Moreover, substantial empirical research has demonstrated that engaging in physical activity can significantly enhance lower-body strength, upper-body strength, and lean body mass in cancer patients undergoing chemotherapy and radiotherapy interventions [97, 98]. A randomized controlled trial was conducted to investigate the impact of resistance training on the maximal muscle strength of postmenopausal breast cancer survivors. The study involved a cohort of 100 participants. The study findings revealed that resistance training positively affected the strength of the upper and lower body in the specific population under investigation [99]. The findings from two meta-analyses suggest that participation in physical activity can positively impact HRQoL, cardiorespiratory fitness, and body composition in individuals who have successfully recovered from breast cancer [100, 101].

Numerous factors have been identified as exhibiting a correlation with the heightened weight and waist circumference commonly observed in individuals who have successfully overcome breast cancer. The variables examined in this study include sedentary behavior [102, 103], psychological distress characterized by symptoms of sadness and worry [104, 105], and premature onset of menopause [106]. Several studies have provided evidence of a substantial correlation between weight gain and the rates of breast cancer recurrence and mortality in a significant portion of breast cancer survivors [107, 108]. Therefore, regular participation in physical activity can provide support to individuals who have been diagnosed with breast cancer, facilitating the control of body weight and improvement of overall health [109].

## Physical exercises and breast cancer mortality

There exists empirical evidence that demonstrates a positive association between engagement in physical exercise and the attainment of a more favorable prognosis, as well as an extended survival period, in individuals who have been diagnosed with breast cancer. Johnsson et al. conducted a study wherein they analyzed a cohort consisting of 847 individuals who had been diagnosed with breast cancer. The age range of the participants encompassed individuals aged 34–87 years. The researchers conducted an observational study on a cohort of individuals who demonstrated the highest level of physical activity. A decrease in overall mortality rates was observed. The study’s results revealed a notable enhancement in the survival rates of individuals who received a breast cancer diagnosis, particularly among women aged 55 years or above. This discovery suggests that the influence of physical activity may be of greater significance for postmenopausal individuals who have been diagnosed with breast cancer [110]. The study conducted by McTiernan et al. has suggested a significant inverse relationship between physical activity and the likelihood of mortality from any cause, suggesting a potential decrease of up to 48%. Furthermore, a similar negative relationship was identified between levels of physical activity and mortality associated with cancer, indicating a potential decrease in risk by 38%. Women who engage in physical activity can exhibit a range of reactions, both before and after receiving a medical diagnosis [111]. Research has demonstrated a positive correlation between the diagnosis of a medical condition in women and their increased vulnerability to the repercussions of participating in physical activity, as compared to individuals who do not have such a diagnosis. The research study results suggest an inverse correlation between increased physical activity levels in women and the likelihood of mortality. The correlation above is notably apparent regarding general mortality, mortality associated with breast cancer, and the capacity to sustain freedom from recurrence. The results of this study contradict the behavior demonstrated by female individuals who engaged in moderate levels of physical activity or experienced a decline in their activity levels within a designated timeframe [112]. Additionally, Palesh et al. conducted a study that found that individuals diagnosed with stage IV cancer at an advanced stage observed a 24% reduction in mortality rate and an extended survival period through implementing moderate physical activity for one hour daily [113]. This discovery juxtaposes individuals who participated in lower levels of physical activity. Lee conducted a recent meta-analysis that revealed a negative correlation between physical activity and all-cause mortality and breast cancer mortality in women engaging in 300 min per week of moderate physical activity [114].

The DELCaP study investigated the association between dietary patterns, physical activity, lifestyle factors, and cancer prognosis. The findings of this study revealed a significant correlation between adherence to minimum guidelines before and following treatment and the subsequent improvement in survival rates and positive prognosis. The correlation above was observed within a sample of 1340 female individuals who received a diagnosis of breast cancer. In addition, the research results have elucidated the influence of physical activity levels on the likelihood of mortality [115]. Before initiating the therapeutic intervention, a comprehensive assessment revealed the existence of a positive association between muscular strength and a favorable prognosis, along with an extended survival period [116]. However, the study did not succeed in establishing any statistically significant correlations between muscle mass and radiodensity. A plethora of extensive documentation exists that delves into the examination of how comorbidities influence the prognosis of breast cancer. This body of research indicates a plausible correlation between the presence of comorbidities and higher rates of mortality [117]. The association between reduced muscular strength in individuals and the simultaneous existence of other medical conditions, coupled with a lack of physical activity, contributes to a less advantageous prognosis and a shorter overall survival duration [118]. The available empirical data indicates a positive association between engagement in physical activity and enhanced prognosis in individuals who have been diagnosed with breast cancer [119]. The studies above offer empirical evidence that supports the implementation of exercise interventions in individuals diagnosed with breast cancer. These interventions have improved overall survival rates, decreased mortality rates, and positively influenced prognosis.

## Conclusion

Experimental studies have demonstrated a positive correlation between engagement in physical activity and a reduced probability of breast cancer incidence. Moreover, there is empirical evidence indicating that participation in physical activity can hinder the advancement of cancer, encompassing the initiation of tumors, dissemination of cancerous cells to distant anatomical sites, and establishment of neovascularization to facilitate tumor expansion. This phenomenon is particularly noteworthy among individuals who have received a diagnosis of breast cancer. Moreover, the incorporation of physical activity has the potential to function as a valuable supplementary intervention in the control of breast cancer, as it possesses the capability to alleviate the negative consequences linked to treatment. This intervention exhibits promise in substantially improving the general well-being of individuals who have been diagnosed with breast cancer while also investigating the complex relationships between quality of life and multiple factors such as fatigue, pain, insomnia, and social and emotional functioning. The current understanding of the correlation between physical exercise and the prevention and treatment of cancer is limited. To establish a clear correlation, it is imperative to possess a comprehensive comprehension of the impact of physical activity on cancer progression, cancer formation, metabolic processes, and immune system functions. It is crucial to acknowledge that physical exercise and lifestyle interventions, although advantageous, do not possess the capacity to provide a cure for breast cancer independently. To enhance the effectiveness of breast cancer management, it is crucial to offer additional support for these interventions. Additional investigation is warranted to delve into the molecular mechanisms that underlie the impacts of vigorous physical activity on breast cancer.

## Data Availability

Not applicable.
